# Genome-wide mapping of DNase I hypersensitive sites in pineapple leaves

**DOI:** 10.3389/fgene.2023.1086554

**Published:** 2023-07-04

**Authors:** Kai Ouyang, Qifu Liang, Li Miao, Zhiliang Zhang, Zhanjie Li

**Affiliations:** ^1^Key Laboratory of Genetics, Breeding and Multiple Utilization of Crops, Ministry of Education, Key Laboratory of Biological Breeding for Fujian and Taiwan Crops, Ministry of Agriculture and Rural Affairs, Fujian Provincial Key Laboratory of Haixia Applied Plant Systems Biology, Center for Genomics and Biotechnology, Fujian Agriculture and Forestry University, Fuzhou, China; ^2^Fujian Key Laboratory of Agro-Products Quality and Safety, Institute of Quality Standards and Testing Technology for Agro-Products, Fujian Academy of Agricultural Sciences, Fuzhou, Fujian, China; ^3^State Key Laboratory of Plant Cell and Chromosome Engineering, Institute of Genetics and Developmental Biology, Innovative Academy for Seed Design, Chinese Academy of Sciences, Beijing, China

**Keywords:** crassulacean acid metabolism, *Ananas comosus*, DNase I hypersensitive sites, cis-regulatory elements, circadian

## Abstract

Pineapple [*Ananas comosus* (L.) Merr.] is the most economically important crop possessing crassulacean acid metabolism (CAM) photosynthesis which has a higher water use efficiency by control of nocturnal opening and diurnal closure of stomata. To provide novel insights into the diel regulatory landscape in pineapple leaves, we performed genome-wide mapping of DNase I hypersensitive sites (DHSs) in pineapple leaves at day (2a.m.) and night (10a.m.) using a simplified DNase-seq method. As a result, totally 33340 and 28753 DHSs were found in green-tip tissue, and 29597 and 40068 were identified in white-base tissue at 2a.m. and 10a.m., respectively. We observed that majority of the pineapple genes occupied less than two DHSs with length shorter than 1 kb, and the promotor DHSs showed a proximal trend to the transcription start site (>77% promotor DHSs within 1 kb). In addition, more intergenic DHSs were identified around transcription factors or transcription co-regulators (TFs/TCs) than other functional genes, indicating complex regulatory contexts around TFs/TCs. Through combined analysis of tissue preferential DHSs and genes, we respectively found 839 and 888 coordinately changed genes in green-tip at 2a.m. and 10a.m. (AcG2 and AcG10). Furthermore, AcG2-specific, AcG10-specific and common accessible DHSs were dissected from the total photosynthetic preferential DHSs, and the regulatory networks indicated dynamic regulations with multiple *cis*-regulatory elements occurred to genes preferentially expressed in photosynthetic tissues. Interestingly, binding motifs of several cycling TFs were identified in the DHSs of key CAM genes, revealing a circadian regulation to CAM coordinately diurnal expression. Our results provide a chromatin regulatory landscape in pineapple leaves during the day and night. This will provide important information to assist with deciphering the circadian regulation of CAM photosynthesis.

## Introduction

Plant growth, development and response to environment rely on precise spatiotemporal transcription of genes. In eukaryotes, gene expression is regulated by orchestrated interaction between transcription factors (TFs) and *cis*-regulatory elements (CREs), generally with the assistance of transcription co-regulators (TCs, (Wray et al., 2003). The interaction of TFs with chromatin requires the interacting regions to be either nucleosome free or undergoing dynamic nucleosome displacements (Henikoff et al., 2009), which consequently cause those regions to be hypersensitive to cleavage by endonuclease DNase I, i.e., DNase I hypersensitive sites (DHSs) (Wu, 1980). Therefore, mapping DHSs has become a powerful “gold standard” approach to identify CREs and TF occupancy in higher organisms (Crawford et al., 2004; Zhang et al., 2014; Mathelier et al., 2015). Combined with high-throughput sequencing, DHS mapping (i.e., DNase-seq) enables the identification of gene regulatory sequences genome wide (Zhang et al., 2014; Vierstra and Stamatoyannopoulos, 2016; Palozola et al., 2019). Recently, several successful applications of DNase-seq have been reported in plants (Zhang et al., 2012a; Zhang et al., 2012b; Sullivan et al., 2014; Cumbie et al., 2015; Qiu et al., 2016; Zhao et al., 2018). In addition, the non-DNase-based method Assay for Transposase Accessible Chromatin sequencing (ATAC-seq) has been applied successfully in plants (Lu et al., 2017; Bajic et al., 2018; Maher et al., 2018).These studies provided insights on the regulatory landscape and transcription factor networks in plant species.

Crassulacean acid metabolism (CAM) plants achieve a higher water use efficiency than C_3_ and C_4_ plants by restricting stomatal opening mainly to the dark and closing stomata during the light. CAM plants can produce comparable above-ground dry-biomass as C_3_ and C_4_ plants, but with only 20% of water required for cultivation (Borland et al., 2009). Elucidating the molecular controls of the CAM pathway may contribute to improving crop for food and energy production in water-limited environments. CAM plants open stomata at night and perform phosphoenolpyruvate carboxylase (PEPC)-mediated CO_2_ fixation during this time, while then the released CO_2_ is refixed by ribulose 1,5-bisphosphate carboxylase/oxygenase (RuBisCO) during the day when the stomata closed. Inverted light/dark stomata movement and diel reprogramming of key enzymes and transporters to sustain the temporal separation of RuBisCO and PEPC have been proposed to be key events of the evolution of CAM photosynthesis (West-Eberhard et al., 2011; Yang et al., 2015). Understanding the circadian regulation of metabolic activities associated with CAM is an important factor for elucidating the CAM pathway and successful application of CAM to crop improvement.

Pineapple [*Ananas comosus* (L.) Merr.] is the most economically important crop possessing CAM photosynthesis. A genomic sequence for pineapple is available (Ming et al., 2015). The availability of high quality genomic and transcriptomic resources and time-course gene expression profiling (Sharma et al., 2017; Wai et al., 2017) makes pineapple an ideal system to study the molecular basis for the circadian regulation of CAM. However, very few information is available to describe the underlying regulatory landscape in pineapple leaves. Previous article reported 1398 transcription factors (TFs) and 80 transcription co-regulators (TCs) in the pineapple genome and evaluated their tissue-specific and diurnal transcript abundance patterns (Sharma et al. (2017). More than 40% of pineapple TFs and TCs displayed diel rhythmic transcript abundance in photosynthetic or non-photosynthetic leaf tissues. Based on transcriptomics patterns and potential functions, candidates related to the circadian rhythm were predicted in the pineapple genome (Sharma et al. (2017). However, the binding sites of these candidate TFs were unknown now, and identifying thousands of TFs in the pineapple genome is a daunting task.

In this study, we developed a simplified and robust method for DNase-seq library construction by introducing a magnetic bead-based fragment selection process. Using this method, we performed genome-wide identification and characterization of DHSs in photosynthetic (green-tip) and non-photosynthetic (white-base) leaf tissues of pineapple. Comparative analysis revealed potential regulatory relationship between clock TFs and CAM photosynthesis genes, and provided potential insights into the diel regulation of CAM photosynthesis genes. These findings help to improve our understanding of the light/dark molecular regulation of CAM photosynthesis in pineapple leaves.

## Materials and methods

### Plant materials

Pineapple cultivar MD-2 plants were grown and maintained in a greenhouse at the Fujian Agriculture and Forestry University (Fujian, China). Young pineapple leaves were collected at 2 a.m. 24 February 2019 and 10 a.m. on 24 February 2019. The time of sunset on 23 February 2019 was 6:00 p.m. and the time of sunrise on 24 February 2019 was 6:31 a.m. The white base and green tip of the leaves were immediately separated and frozen in liquid nitrogen for the DNase-seq experiment. Three biological replicates of each leaf tissue sample were prepared.

Seeds of *Arabidopsis thaliana* Col-0 were germinated in one-half–strength Murashige and Skoog medium. The seedlings were grown under 16-h light/8-h dark cycles at 22°C for 2 weeks, and the leaf tissues were then collected and immediately frozen in liquid nitrogen for DNase-seq analysis.

### Nucleus isolation, DNase I digestion and sequencing libraries construction

The nucleus isolation process was performed according to a method published previously with minor modifications (Zhang and Jiang, 2015). Briefly, leaf samples were ground into fine powder in liquid nitrogen and transferred into a 50 mL corning conical tube; then, the powder was suspended in nuclear isolation buffer (NIB; 20 mM Tris-HCl, 50 mM EDTA, 5 mM spermidine, 0.15 mM spermine, 0.1% mercaptoethanol, 40% glycerol at pH 7.5), washed with nuclear wash buffer (NWB; adding 0.5% Triton X-100 into NIB), and finally resuspended in nucleus digestion buffer (NDB; 10 mM Tris-HCl, 10 mM NaCl, 3 mM MgCl_2_, pH 7.4).

The suspended nuclei were digested with DNase I for 10 min at 37°C. The use of a series of DNase I concentrations is recommended to optimize DNase I digestion. Then, the reactions were stopped by adding 50 mM EDTA. The digested chromatin was incubated with proteinase K at 55°C for 1 h. DNA was isolated using phenol-chloroform extraction, ethanol precipitated and finally dissolved in ddH_2_O. The DNA samples were first subjected to size selection by magnetic beads to remove fragments larger than 1 kb. The remaining smaller DNA fragments were used to construct DNase-seq libraries using the NEBNext Ultra DNA Library Prep Kit for Illumina (New England Biolabs, E7370), and then a second size selection for insertions less than 200 bp was carried out. The DNase-seq libraries were paired-end sequenced using Illumina Hiseq2500.

### DHSs identification and genome-wide annotation

The DNase-seq reads were aligned to the latest version of pineapple genome (https://www.life.illinois.edu/ming/downloads/) using the BOWTIE program with a 1-bp mismatch allowed (Langmead et al., 2009). Only the reads mapped to a unique position of the pineapple genome were used for further analysis. We used Picard CollectInsertSizeMetrics (http://broadinstitute.github.io/picard) to assess the distribution of fragment insertions. The reads derived from fragments <= 125 bp were selected for peak calling. The DHS peaks were identified using the MACS2 program with *q-value* <= 0.05 (Zhang et al., 2008). Three biological replicates were analyzed respectively and the pineapple final peaks were generated using IDR process with threshold of 0.05 (Li et al., 2011). Peak annotation was performed with the ChIPseeker package according to pineapple gene annotation information (Yu et al., 2015). The promoter region was separated into −2000 to −1000 bp and −1000 to 0 bp relative to the transcription start site (TSS). The downstream regions were separated into 0–1000 bp and 1000–2000 bp downstream of the transcription end site (TES). Then, the remaining regions between adjacent genes were defined as distal intergenic regions. The peaks were visualized using the Integrative Genomics Viewer (Robinson et al., 2011; Thorvaldsdottir et al., 2013). The read densities were calculated by reads per 10 bp and then used to plot profiles using Deeptools (Ramirez et al., 2016).

### Preferentially accessible DHSs, differentially expressed genes and GO function analysis

The preferentially accessible DHSs (differential DHSs, DDHSs) were identified as the method previously described in Sijacic et al. (2018); Bubb and Deal (2020). DHSs identified in each tissue were merged to create a union set of DHSs. The read number for each tissue in the union DHSs was counted using BEDTools (Quinlan and Hall, 2010).Three replicates from each tissue were counted, and the counts were processed using DESeq2 package in R (Love et al., 2014). Those DHSs that had a fold change> =2 and an adjusted *p*<= 0.05 for a specific tissue were identified as tissue preferentially accessible DHSs.

The RNA-seq data were from the previous study (Ming et al., 2015). For differentially expressed genes (DEGs) identification, the RNA-seq data were analyzed using hisat2 (Kim et al., 2019) and featureCounts (Liao et al., 2014), and then read counts from each replicates were also processed using DESeq2 to found DEGs. The GO information of pineapple genes was retrieved from Phytozome (https://phytozome.jgi.doe.gov). GO enrichment analysis was performed using clusterProfiler package in R (Yu et al., 2012), and GO terms that had FDRs of 0.05 or less were considered significant. Gene expression heatmaps were obtained using Genesis software (Sturn et al., 2002).

### Motif occurrence and regulatory networks construction

For motif occurrence analysis, the DNA sequences from the DHS peaks were firstly obtained using BEDTools (Quinlan and Hall, 2010), and the motif occurrences within the DHSs were analyzed using the FIMO tool (Grant et al., 2011). Those locations with *p* < 1e-04 were considered as significant motif occurrences. For a given TF, whose predicted binding sites were identified in the DHSs nearest to the TSS of another gene, then the nearest gene was considered as a putative regulatory target of the TF. The regulatory networks were conducted as previously described by (Sullivan et al., 2014). Briefly, an edge (TF-to-gene interaction) will be created when a TF binding site occurred within a DHSs which nearest to the TSS of the target gene. The regulatory network connections between genes were visualized by Cytoscape v.3.4.0 (https://cytoscape.org/).

## Results

### Development of a simplified DNase-seq protocol

**FIGURE 1:**
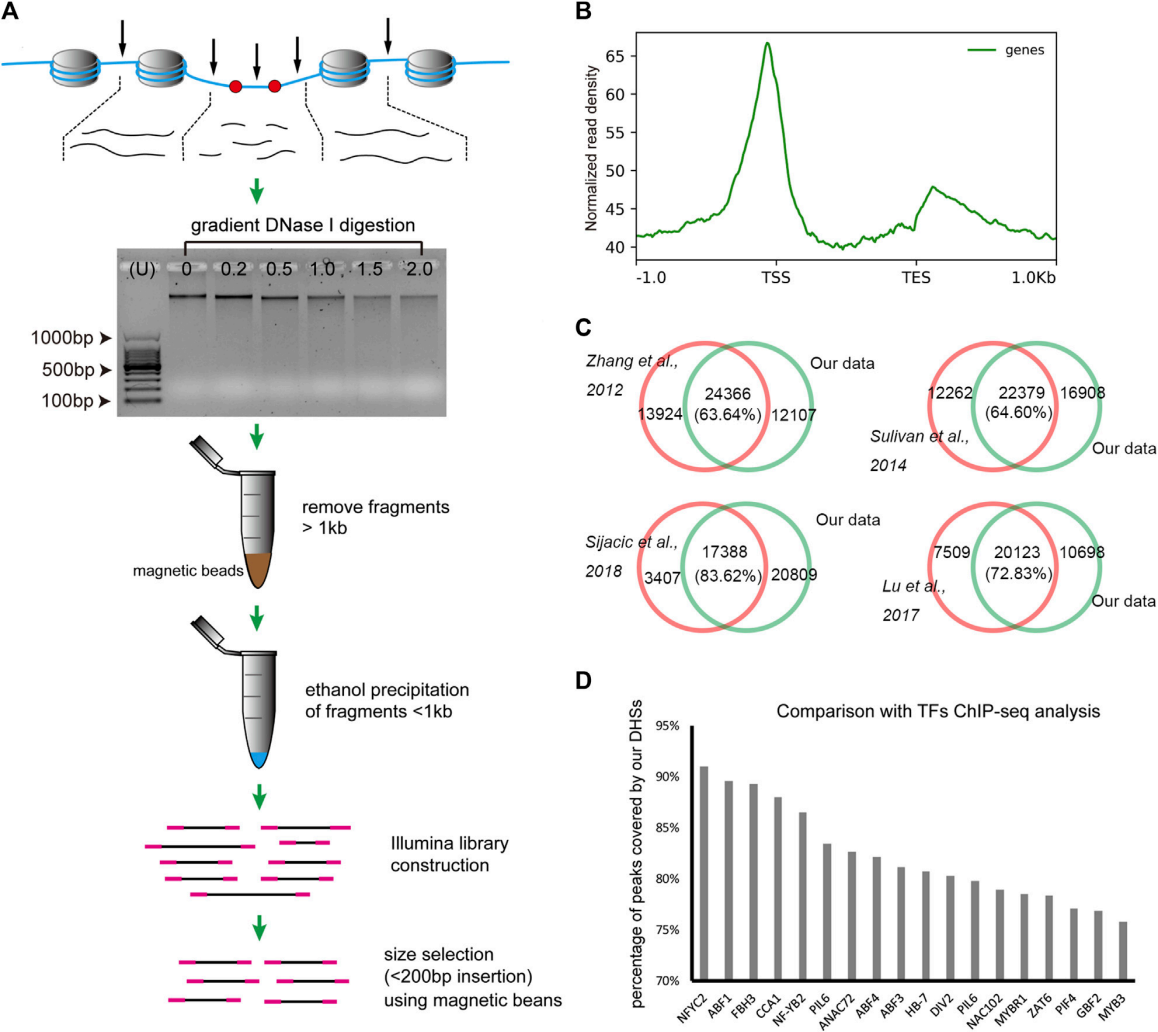
The simplified DNase-seq protocol developed in this study. **(A)**. The illustration of the simplified DNase-seq library construction. **(B)**. The distribution of DNase-seq reads revealed peaks at the TSS and TES regions. **(C)**. Venn diagram showing the significant overlapping of DHSs between our and previous results (Zhang et al., 2012b; Sullivan et al., 2014; Lu et al., 2017; Sijacic et al., 2018). **(D)**. Percentage of peak coverage between DHSs in our study and TF peaks identified in previous ChIP-seq analysis (Song et al., 2016).

In this study, we firstly developed a simplified DNase-seq method based on the traditional double-hit DNase-seq (Sabo et al., 2006) method in order to obtain longer sequence reads, and replaced the gel-based fragment selection process by a magnetic beads-based method to significantly shorten the library construction time. The procedure was illustrated in Figure 1A, after gradient DNase I digestion, the chromatin related DNA were extracted and visualized on the agarose gel, then the large DNA fragments (about more than 1 kb) were removed and the remaining smaller fragments were used for library construction, finally the fraction of fragments with insertion less than 200 bp was separated using magnetic beads for sequencing.

To assess the validation of the method, we firstly developed DNase-seq libraries for *Arabidopsis* leaves and compared with previous *Arabidopsis* leaf data derived from different approaches (Zhang et al., 2012b; Sullivan et al., 2014; Lu et al., 2017; Sijacic et al., 2018). After removing multi-mapped and organelle-derived reads, we finally obtained 46.1, 46.1 and 48.2 million clean reads ( ≤ 125bp) for DHS peak calling in each of the three biological replicates. The pearson’s correlation coefficient of the three biological replicates were ranged from 0.95 to 0.96, indicating highly reproducible results (Supplementary Figure S1). Reads distribution analysis demonstrated that these reads were enriched around the transcription start site (TSS) and transcription end site (TES) and eclipsed in the gene body (Figure 1B), which is consistent with the previous DHSs analysis studies (Zhang et al., 2012b; Sullivan et al., 2014; Sijacic et al., 2018). Then we conducted DHS peak calling using MACS2 and merged all peaks from three biological replicates, then totally 38,691 peaks were kept as the final peak for our data set. Comparing with the 20,795 to 38,290 DHS peaks identified in previous studies in *Arabidopsis* (Zhang et al., 2012b; Sullivan et al., 2014; Lu et al., 2017; Sijacic et al., 2018), we found that 63.64%–83.62% of those published DHSs were recalled in our data (Figure 1C). Moreover, we obtained the genome-wide binding sites of 18 transcription factors identified using ChIP-seq in *Arabidopsis* seedlings (Song et al., 2016) (http://plantregmap.cbi.pku.edu.cn/). Strikingly, 75.8%–91.0% of these TFs binding sites were overlapped with our DHS peaks (Figure 1D). Taken together, the results indicate that the simplified DNase-seq method is reliable for genome-wide DHSs detecting in plant species.

### Genome-wide identification of DHSs in pineapple leaf tissues

**FIGURE 2:**
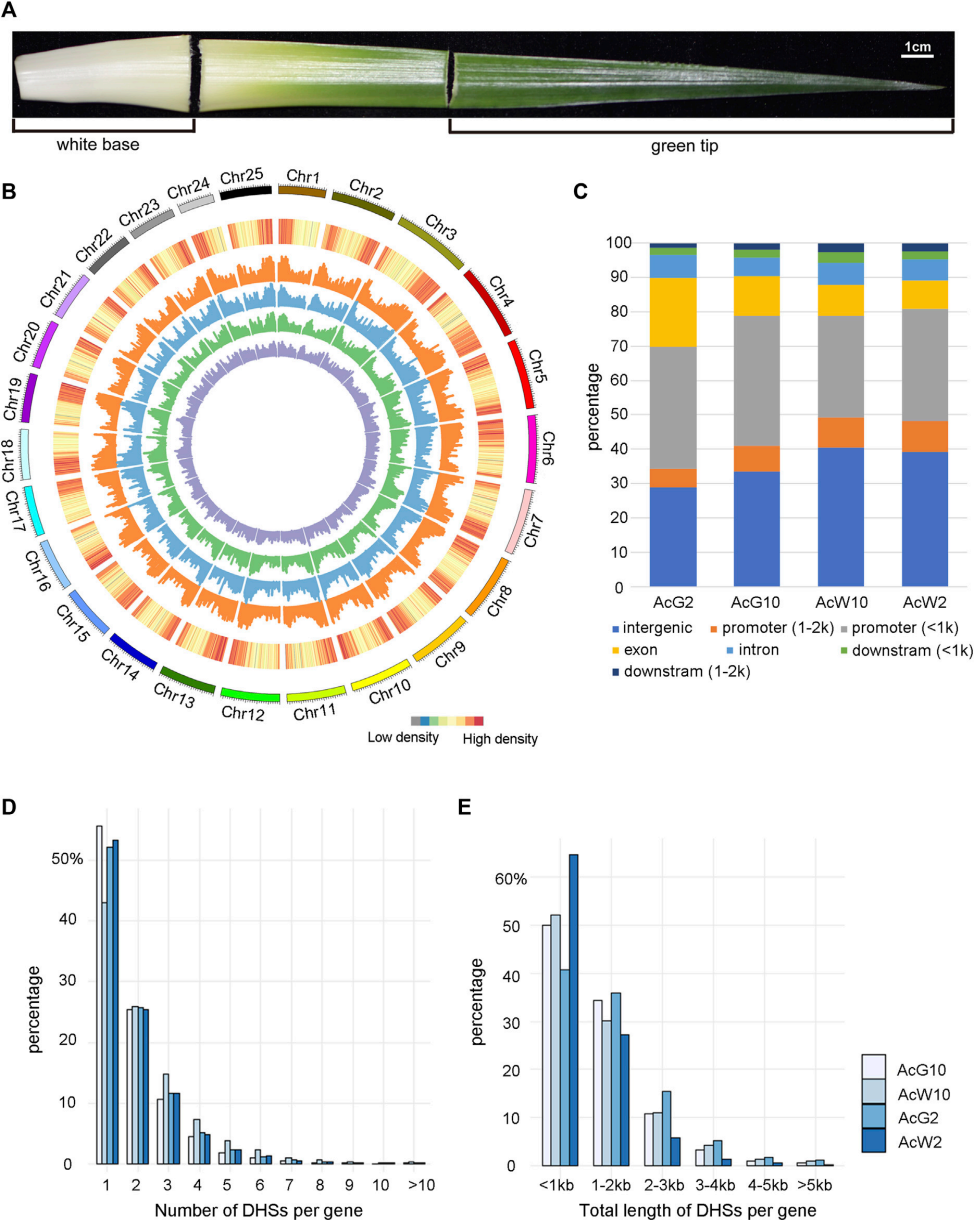
Genome-wide distribution of DHSs and annotation based on location. **(A)**. Pineapple leaf sections were collected for analysis. Photosynthetic (green tip) and non-photosynthetic (white base) areas are indicated. **(B)**. The genome-wide distribution of DHSs across 25 pineapple chromosomes. From outside to inside, the circles represented the chromosome, gene density and DHS abundance in four pineapple leaf tissues (orange for AcW10, blue for AcW2, green for AcG10, and purple for AcG2). **(C)**. The distribution of DHSs relative to different gene regions in each sample. **(D)**. The distribution of genes with different number of DHSs in the four pineapple tissues. **(E)**. The distribution of genes with different length of DHSs in the four pineapple tissues.

To investigate the genome-wide regulatory landscape in pineapple leaves during day and night, we selected photosynthetic (green-tip) and non-photosynthetic (white-base) leaf tissues at 2a.m. and 10a.m. to construct four DNase-seq libraries, green-tip at 2 a.m. (AcG2) and 10 a.m. (AcG10) and white-base at 2 a.m. (AcW2) and 10 a.m. (AcW10) (Figure 2A), each with three biological replicates. Finally, we generated from 17.2M to 39.5M uniquely mapped reads ( ≤ 125bp) in each of the replicate for AcG2, AcG10, AcW2 and AcW10, respectively (Supplementary Table S1). The pearson’s correlation coefficient of replicates were ranged from 0.97 to 0.99 (Supplementary Figure S2), indicating high reproducibility across replicates. And the read distribution were also enriched around the TSS and TES like Arabidopsis data (Supplementary Figure S3). The DHSs peak calling procedure was the same with *Arabidopsis* data analysis. In addition, to obtain more repeatable peaks, we used the IDR method with threshold of 0.05 to obtain reliable peaks from three biological replicates. Finally, we identified a total of 33340, 28753, 29597, 40068 peaks in AcG2, AcG10, AcW2, and AcW10, respectively (Supplementary Datasets S1), which covered 4.8%–8.0% of the pineapple genome.

The distribution assay across the genome showed that DHSs were enriched in the chromosome distal regions and were depleted from regions of gene deserts, which are usually the centromeric or pericentromeric regions (Figure 2B). These is consistent with trends observed in other plants (Zhang et al., 2012b; Sullivan et al., 2014; Qiu et al., 2016; Rodgers-Melnick et al., 2016). Then we examined the location of DHSs relative to genes, overall similar distribution of DHSs both in number (Figure 2C) and length (Supplementary Figure S4) were identified from the four samples. Approximately 40% of the DHSs were located within 2 kb upstream of a TSS, which is usually defined as a promoter region. Interestingly, the majority of those promoter DHSs (76.93%–86.64%) were located within 1 kb upstream of the TSS, indicating a coding region proximity trend for the promotor regulatory elements in pineapple genome. Approximately 15%–20% of the DHSs were mapped in gene-body regions (intron, exons). Furthermore, approximately 30%–40% of the DHSs were cataloged as intergenic regions (no genes within ±2 kb flanking the DH sites).

In order to analysis the relationship of DHSs and functional genes in pineapple, we associated each DHS with the nearest gene based on its distance from the nearest TSS. As a result, fifty-eight to sixty-six percentage of the whole genomic genes were found to be associated with at least one DHS in four tissues. Interestingly, about 50% of the genes were associated with only one DHS, and another 25% were associated with two DHSs (Figure 2D). Similarly, the distribution of total length of DHSs assigned to each gene was also analyzed. Consistently, majority of (about 50%) the genes were assigned with DHSs less than 1 kb and another 30% were assigned with DHSs range from 1–2 kb in length (Figure 2E). These results indicated that most of the pineapple genes were associated with less and shorter DHSs in leaf tissues.

### More intergenic DHSs nearby TFs/TCs indicating a more complex regulatory context around them

**FIGURE 3:**
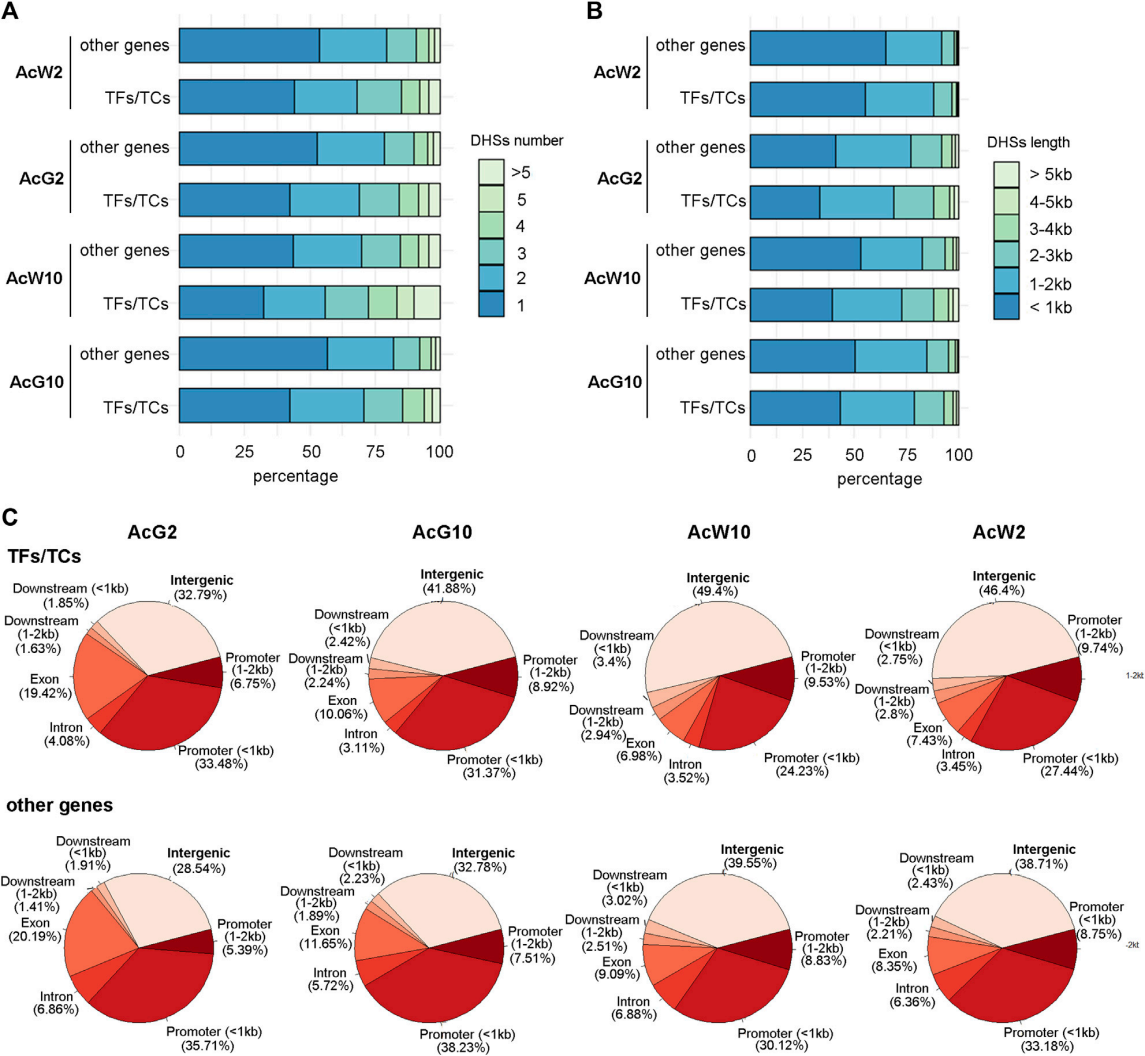
The features of DHSs assigned to TF/TC genes and other functional genes in four pineapple tissues. **(A)**. The comparison of DHS numbers of per gene between TF/TC and other functional genes. **(B)**. The comparison of total DHS length of per gene between TF/TC and other functional genes. **(C)**. The distribution of DHS locations around TF/TC and other functional genes.

Previous study totally annotated 1,398 TFs from 67 TF families and 80 TCs from 20 TC families in pineapple genome (Sharma et al., 2017). To investigate the DHSs features of TF and TC genes, we separate the TFs/TCs from other functional genes and calculated the number and total length of DHS around them, respectively. The proportion of TFs/TCs with more than 1 DHSs around them was nearly 10% percent greater than those of other functional genes (Figure 3A). Consistently, the proportion of TF/TC genes associated with DHSs longer than 1 kb was greater than those of other functional genes (Figure 3B). We further compared the distribution of DHSs based on genomic locations between TFs/TCs and other functional genes. The results indicated that relative to other functional genes, TFs/TCs exhibited more proportion of DHSs located in the intergenic regions (upstream or downstream 2 kb away from genes; Figure 3C). Furthermore, we were curious that whether the extra proportion of DHSs detected in the number or length analysis above was coming from intergenic regions. We separated the top 50% DHS-numbered TFs in each tissue, and the location distribution analysis indicated that 38%–56% of the DHSs assigned to these TFs were located in the intergenic regions (Supplementary Table S2), which is significantly higher than the level of 35%–46% in total TF/TC genes (Supplementary Table S2). A similar result was also obtained during the analysis of top 50% of TFs which having the most length of the DHSs (Supplementary Table S2). These results indicated that more and longer DHSs were around TF/TC genes comparing to other functional genes, and most of them were located in intergenic regions. Thus, we inferred that the TFs/TCs located in a more complex regulatory context in pineapple genome.

### Preferentially accessible DHSs and the coordinately changed genes in pineapple leaves

**FIGURE 4:**
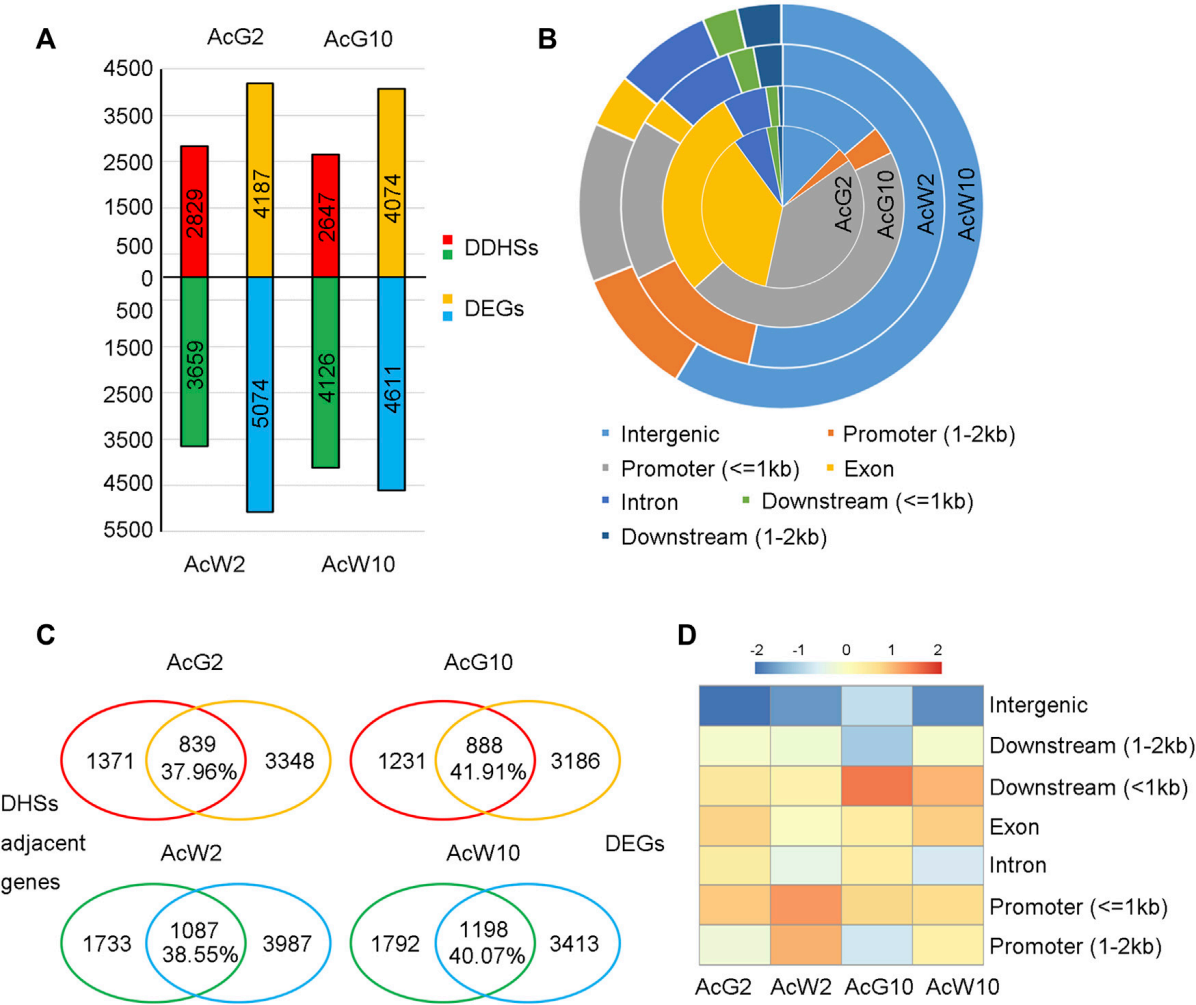
Identification of tissue preferentially accessible DHSs and the coordinately changed genes in four pineapple leaf tissues. **(A)**. The identification of tissue preferentially accessible DHSs (Differential DHSs, DDHSs) and differentially expressed genes (DEGs). **(B)**. The location distribution of DDHSs identified in four tissues. **(C)**. The comparative assay to find coordinately changed genes (CCGs) in four tissues. The overlapped genes were considered as CCGs, the percentage indicate the ratio of CCGs to total genes which were assigned with any DHS. **(D)**. Ratio heatmap of overlapped genes associated with DHSs located at distinct regions.

To gain the tissue preferential regulatory landscapes in photosynthetic leaf tissues during light/dark cycling, we conducted DHS comparisons between the four samples according to the method mentioned in previous article (Sijacic et al., 2018; Bubb and Deal, 2020). Firstly, a set of union DHSs were defined by merging all of the DHSs form four tissues, resulting in totally 53,222 peaks. Then, quantitative comparisons were made between samples to looking for those union regions with differential accessibility using DEseq2. DHS union regions with fold change>=2 and *p*-value ≤ 0.05 were considered as different DHSs (DDHSs, Supplementary Datasets S2). In total, we identified 6,773 DDHSs between AcG10 vs. AcW10, with 2,647 DDHSs preferentially accessible in AcG10 and 4,126 DDHSs enriched in AcW10 (Figure 4A). In addition, 6,488 DDHSs were found between AcG2 vs. AcW2, in which 2,829 DDHSs preferentially accessible in AcG2 and 3,659 DDHSs enriched in AcW2 (Figure 4A). These preferentially accessible DHSs were considered as tissue-specific DHSs.

The distribution of these tissue-specific DHSs based on genomic locations were shown in Figure 4B. Interestingly, DHSs preferentially accessible in photosynthetic leaf tissues (AcG2 and AcG10) were mostly located in the promoter regions, especially in 1 kb upstream of the TSS. In contrast, the non-photosynthetic leaf tissues (AcW2 and AcW10) specific DHSs were mainly located in the distal intergenic regions. Fisher’s exact test indicated that the number of DDHSs nearest to TF genes were significantly higher in non-photosynthetic leaf tissues (AcW2, 323 TFs vs. 3659 in total; AcW10, 393 TFs vs. 4126 in total) than those in photosynthetic leaf tissues (AcG2, 213 TFs vs. 2829 in total; AcG10, 184 TFs vs. 2647 in total) with *p*-value <0.05 and odds radio ranged from 1.19 to 1.41.

To functionally annotate these tissue-specific DHSs, we performed RNA-seq analysis by comparing gene transcription between photosynthetic and non-photosynthetic leaf tissues. A total of 9,261 and 8,685 differentially expressed genes (DEGs) were identified in AcG2 vs. AcW2 and AcG10 vs. AcW10 comparison, respectively (fold change>=2, *p*-value ≤ 0.05) (Figure 4A; Supplementary Datasets S2). As expected, GO enrichment analysis indicated that the DEGs preferentially expressed in photosynthetic leaf tissues (AcG2 and AcG10) were enriched in photosynthesis related pathways, while DEGs preferentially expressed in non-photosynthetic leaf tissues (AcW2 and AcW10) were enriched in metabolism (Supplementary Figure S5).

In order to investigate whether the tissue-specific DHSs were coordinately changed with the gene expression, we assigned each tissue-specific DHS to the closest TSS and examined the overlap between the assigned genes and DEGs. As a result, from 37.96% to 41.91% of the tissue-specific DHS adjacent genes were also differentially expressed in that tissue (Fisher’s exact test, *p*-value <0.05 and odds radio ranged from 1.90 to 2.59, Figure 4C), the overlapped genes were considered as the coordinately changed genes (CCGs). GO enrichment analysis also indicated that the CCGs in photosynthetic leaf tissues (AcG2 and AcG10) were enriched in photosynthesis related pathways (Supplementary Figure S6). Moreover, it is interesting that we respectively found 54, 47, 116, 121 TF/TCs in the CCGs of each tissue (AcG2,839; AcG10,888; AcW2,1087; AcW10,1198), including several circadian clock related genes, such as *CCA1*, *LHY1*, *RVE1*, *PRR2*, *PRR5*, *PIF3*, *PIF5*, *CDF2*, *CDF3*. These results suggested that the tissue-specific DHSs potentially contained *cis*-regulatory elements (CREs) for regulation of genes in circadian and photosynthesis related pathways.

Furthermore, detailed analysis of the distribution of coordinately changed genes (CCGs) according to DHS location indicated that DHSs located nearby the gene, especially within 1 kb upstream or downstream, can easily result in a higher proportion of CCGs (Figure 4D). These results suggested that the gene-nearby DHSs had more direct regulatory effects on gene expression alteration, while the distal DHSs had weak correlation with gene expression changing.

### Light/dark induced regulatory network in pineapple photosynthetic tissues

**FIGURE 5:**
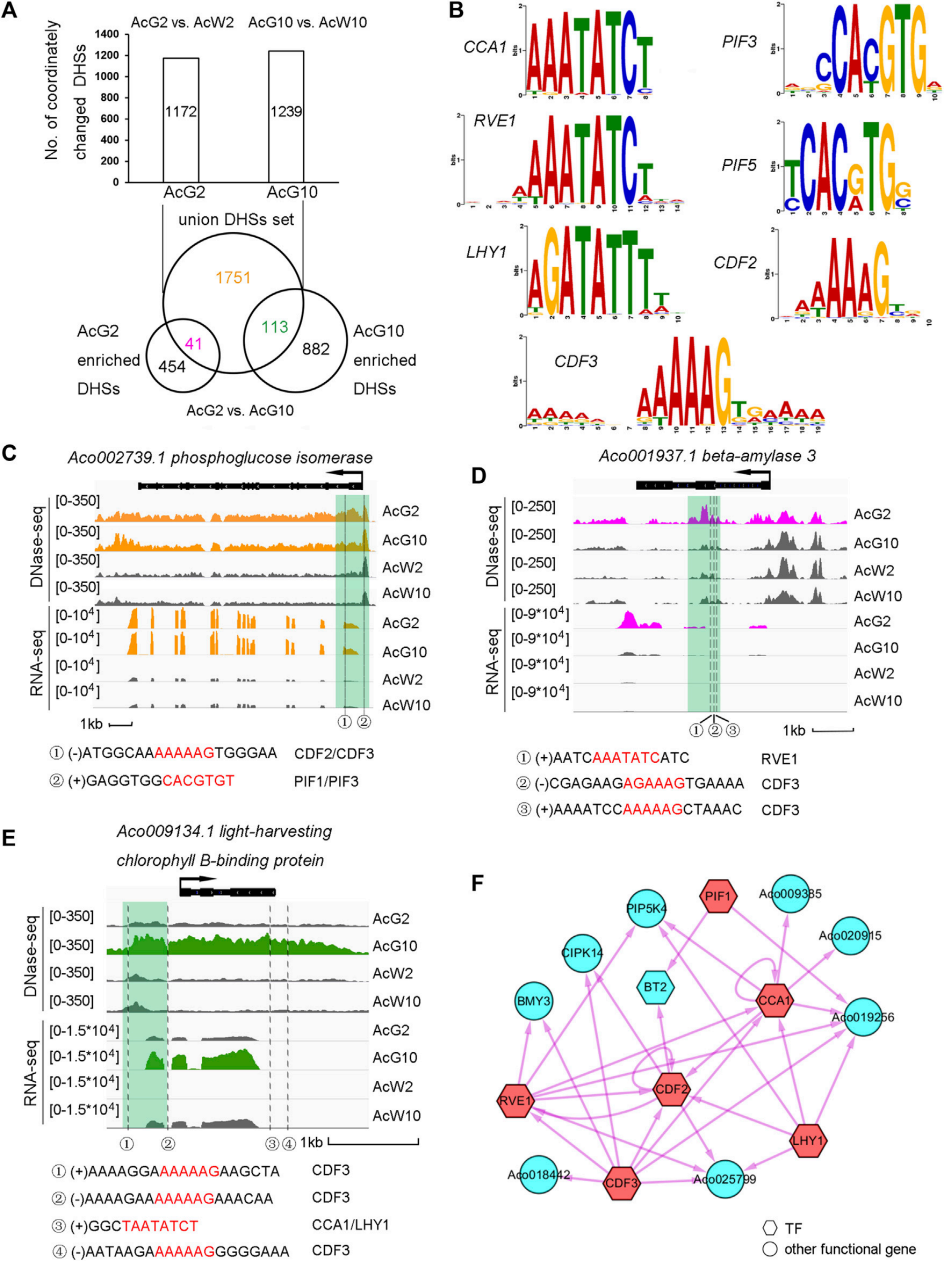
Regulatory landscape between clock TFs and phtotosynthesis related genes in the photosynthetic tissue. **(A)**. Dissection of photosynthetic preferentially accessible DHSs assigned to the CCGs into AcG2-specific, AcG10-specific and common accessible DHSs. **(B)**. The motifs of 7 clock related TFs selected for further analysis **(C–E)**. The illustration of three typical genes which coordinated regulated by three types of DHSs (C, common accessible DHSs; D, AcG2-specific DHSs; E, AcG10-specific DHSs). The profile of tissue-specific expression pattern was consistent with tissue-specific DHSs nearby the gene. The clock related motif occurrences in the DHSs were listed below. **(F)**. The regulatory network between the CCGs with only AcG2-sepcific DHSs. The clock related TFs were colored in red and the potential targets in blue. TFs were showed as hexagons and other functional genes as circles.

To further explore the dynamic regulation in photosynthetic tissues induced by light/dark cycling, we isolated the 1172 and 1239 photosynthetic preferentially accessible DHSs annotated to the CCGs in AcG2 (839) and AcG10 (888), obtaining a union set of totally 1905 DHSs (Figure 5A). When comparing the union DHSs set with AcG2-or AcG10-preferentially accessible DDHSs, which resulting from direct DHSs comparison between AcG2 and AcG10 (Supplementary Datasets S2), we divided the 1905 union DHSs into three parts, including 41 DHSs more accessible in AcG2 (AcG2-specific), 113 DHSs more accessible in AcG10 (AcG10-specific), and 1751 DHSs both accessible in AcG2 and AcG10 with no difference (common). The GO analysis of genes annotated to the 41 and 113 DHSs indicated that the genes with AcG2-specific DHSs are functionally related to polysaccharide catabolic while those genes with AcG10-specific DHSs were enriched in phtotosynthesis pathway (Supplementary Figure S7).

In order to investigate the potential regulatory network between TFs and their target genes. We isolated 82 TFs from the total 1313 CCGs from AcG2 and AcG10, and interestingly found so many circadian clock related TFs were included, such as *CCA1*, *LHY1*, *RVE1* etc. (Supplementary Datasets S3). Finally, seven core clock related TFs, *CCA1*, *CDF3*, *PIF1*, *LHY1*, *RVE1*, *CDF2*, *PIF3*, which all have homologous genes in *Arabidopsis*, were selected for further analysis. The motifs of the selected TFs were showed in Figure 5B. Then, we scanned these 7 clock motif sequences in the DHS regions from AcG2-specific, AcG10-specific, and common accessible (AcG2 and AcG10) datasets. The nearby genes annotated to the DHSs which a TF motif was occurred in were considered as the potential regulatory targets of that TF. For insurance, DHSs located within 2 kb upstream or downstream of the annotated genes were used for analysis. Finally, we found 2522 TF motif occurrences in the DHSs around 911 potential regulatory target genes (Supplementary Datasets S3). Interestingly, there were another 59 TF genes in the potential regulatory targets of these 7 clock TFs, and 42 (71%) of which exhibited diurnal cycling expression pattern in photosynthetic leaves (Sharma et al., 2017). These results indicated that the strategy for identification of regulatory targets of TF genes in this study is reliable.

Furthermore, we observed a lot of phtotosynthesis related genes in the potential regulatory targets of the 7 clock TFs (Supplementary Datasets S3). Here we took three typical examples for detailed description. Firstly, *Aco002739.1, phosphoglucose isomerase*, which exhibited higher expression levels in photosynthetic leaf tissues (AcG2 and AcG10) than non-photosynthetic leaf tissues (AcW2 and AcW10). Consistently, we found a DHS at the 5′UTR region which were common more accessible in AcG2 and AcG10 than AcW2 and AcW10. And we interestingly identified two motif occurrences of *CDF2*/*CDF3* and *PIF1*/*PIF3* in this DHS, indicating this gene might be a potential regulatory target of *CDF2*/*CDF3* and *PIF1*/*PIF3* (Figure 5C). Secondly, *Aco001937.1, beta-amylase 3*, exhibited a significantly high level of gene expression only in the AcG2. A AcG2-specific DHS was found in the exon region of this gene, in which three motif occurrences of *RVE1* and *CDF3* were detected (Figure 5D). Thirdly, *Aco009134.1, light-harvesting chlorophyll B-binding protein*, showing a preferentially high expression level only in AcG10, was found a AcG10-specific DHSs at the promoter and downstream region. We detected four motif occurrences of *CCA1*/*LHY1* and *CDF3* in the these DHSs (Figure 5E). These results indicating that these core circadian related TFs might participate in regulation of phtotosynthesis related genes expression in green leaf tissues during the day/night cycling.

To clearly illustrate the regulatory relationship between the clock TFs and the potential target genes, we constructed three regulatory networks among the three parts of CCGs, which were nearby occupied by AcG2-specific DHSs, AcG10-specific DHSs, or common accessible DHSs both in AcG2 and AcG10. For brevity, only the TF-to-TF relationship were showed in the regulatory network of genes harboring common accessible DHSs (Figure 5F; Supplementary Figure S8, Supplementary Datasets S3). As a result, we not only found several inter-regulation and self-regulation between clock TFs, but also found many phtotosynthesis related genes were the potential regulatory targets of clock TFs, such as *phosphoglucose isomerase* (*PGI*), *phosphoglucomutase* (*PGM*), *UDP-glucose pyrophosphorylase* (*UPG*), and so on. Most excitingly, we found that several clock related motifs also occurred in the DHSs around CAM pathway genes, such as *PEPC*, *MDH*, *beta-CA* (Supplementary Datasets S3).

### The coordinated regulation of key CAM genes in pineapple leaves

**FIGURE 6:**
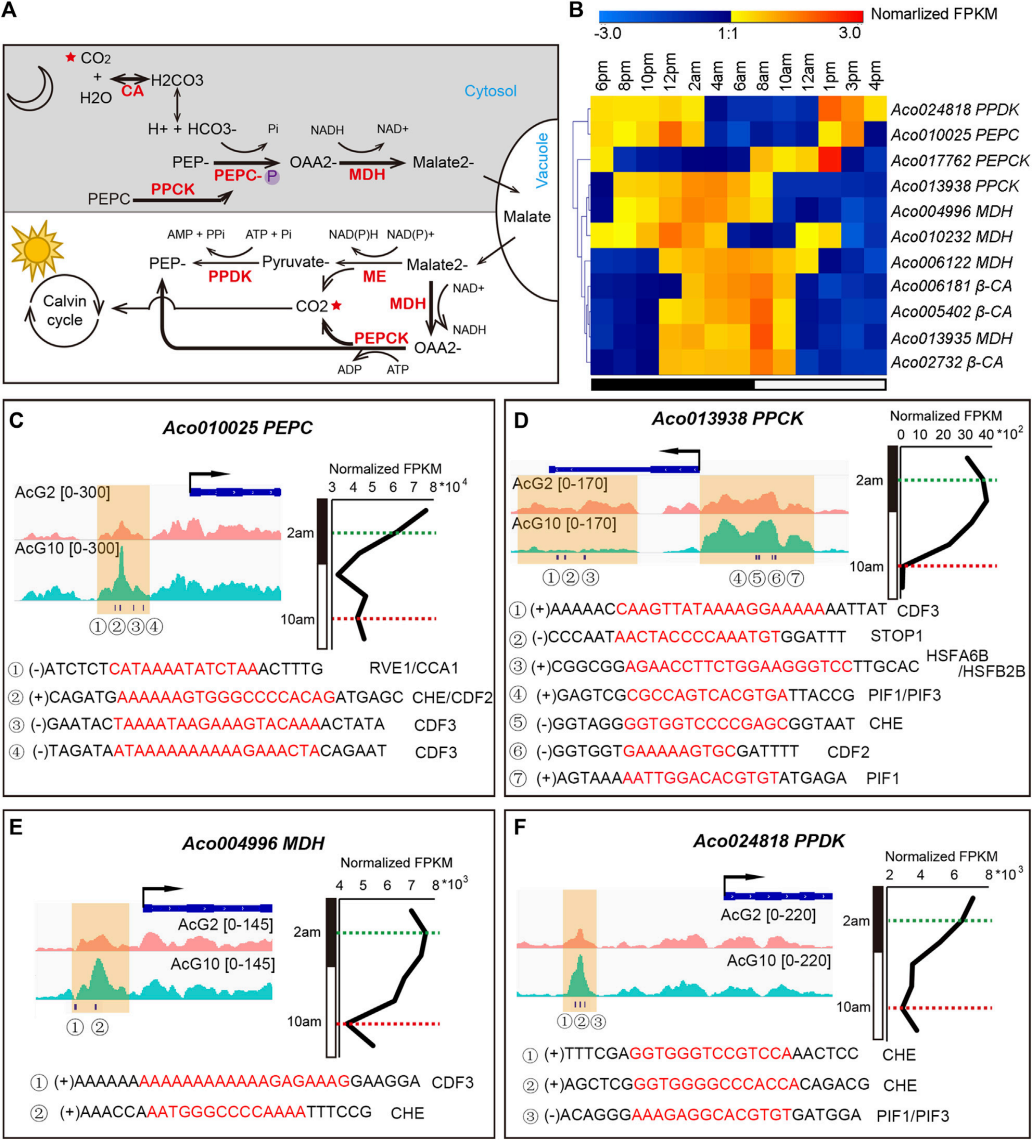
The coordinated regulation of the key CAM genes in pineapple leaves. **(A)**. Overview of the CAM pathway, including carboxylation (at night) and decarboxylation (in the day) processes. The main CO_2_ metabolism processes in pineapple were highlighted in bold arrows, and key enzymes are shown in red. **(B)**. The diurnal expression patterns of 11 CAM enzyme encoding genes during the day/night cycling. **(C–F)** Visualization of DHSs and circadian related TF motif occurrences around the CAM genes *PEPC* **(C)**, *PPCK* **(D)**, *MDH* **(E)** and *PPDK* **(F)**. The gene expression pattern over a 24-h cycle was shown on the right. The timepoints at 2 a.m. and 10 a.m. were indicated by green and red dashed lines, respectively. The DHSs identified from AcG10 and AcG2 were shaded in yellow. The circadian related TF motifs identified in each DHS were listed below.

The CAM pathway is a metabolic adaptation to arid environments and is particularly noteworthy for its high water-use efficiency. The light/dark cycling of CO2 metabolism in the CAM pathway depends on the coordination of several key enzymes (Figure 6A). Based on homology analysis of known C3 and C4 photosynthesis genes, 38 putative enzyme encoding genes in CAM pathway were identified in pineapple genome (Ming et al., 2015). Of these, 11 genes showed diurnal expression patterns in the green tips but either were inactive or had low levels of expression in white bases (Figure 6B; Supplementary Figure S9). And we are curious that whether these diurnally expressed genes were regulated by circadian related genes. Thus, we searched 12 clock regulation related motifs in the DHSs around the 11 diurnally expressed CAM genes, and surprisingly found that almost all of these CAM genes have at least one clock motif in the DHSs nearest to them (Supplementary Datasets S4). The 12 clock related motifs include 7 core clock TFs (*CDF2*, *PIF1*, *CDF3*, *RVE1*, *CCA1*, *PIF3*, *LHY1*), and another 5 important TFs being observed with diurnal expression patterns in pineapple leaf tissues previously (*STOP1*, *HSFA6B*, *HSFB2B*, *CHE*, *LUX*)(Sharma et al., 2017).

Four genes, *Aco013938* (*PPCK*), *Aco024818* (*PPDK*), *Aco004996* (*MDH*) and *Aco010025* (*PEPC*), are key points of regulation in CAM pathway and showed distinct expression patterns (∼1.5 times FPKM difference) at 2 a.m. and 10 a.m. (Figure 6B; Supplementary Figure S9). Thus, we selected them for detailed analysis. For accuracy, the DHSs located within 2 kb upstream or downstream of the genes were shown in Figures 6C–F. Obviously, there are distinct accessible DHSs in the promoter region of the four genes and lots of clock motifs were found in the tissue-specific DHSs. *Aco010025* is one of the three candidate genes encoding phosphoenolpyruvate carboxylase (PEPC, EC 4.1.1.31) in pineapple genome, and serves as the key enzyme that fixes CO2 to phosphoenolpyruvate (PEP) in the cytosol during the nighttime (Borland et al., 2014). *Aco010025* shows a day/night changing expression pattern, from a high expression level at night to continuous downregulation and keeping a low expression level in the day (Figure 6C). Consistently, we found a significantly differential DHSs at the promoter region of this gene, which exhibited more accessible at 10 a.m. than at 2 a.m. (Figure 6C). Examining of clock related motifs in this DHS resulted in identification of co-occurrence of *CCA1*, *RVE1*, *CHE*, *CDF2* and *CDF3*. These results indicated that these clock related TFs might be involved in coordinating the dynamic expression of the *PEPC* in pineapple leaves.

*Aco013938* is one of the two candidate genes encoding phosphoenolpyruvate carboxylase kinase (PPCK) in pineapple genome, which is another key enzyme in CAM pathway to regulate the activity of PEPC through catalyzing its phosphorylation state, and is thought to be tightly regulated by the circadian clock in CAM plants (Hartwell et al., 2016). *Aco013938* exhibited the highest expression at dawn (4 a.m.–6 a.m.), and its expression then decreased sharply to the lowest level at 10 a.m., resulting in more than 35-fold difference in expression between 2 a.m. and 10 a.m. (Figure 6D). We found 8 DHSs which nearest to the TSS of *Aco013938*, and the clock related motifs can be identified in 5 of the 8 DHSs. Interestingly, two adjacent DHSs were found in the promoter region and gene body region nearby TES, respectively. The one located in the promoter region showed more accessible at 10 p.m. than 2 a.m., and we found several motif occurrences in it, including *PIF1*, *PIF3*, *CHE* and *CDF2*. However, the other one located nearby TES was more accessible at 2 a.m. than 10 a.m., and the motifs of *CDF3*, *STOP1* and *HSFA6B*/*HSFA2B* were found in it. These results indicated that the clock related TFs must be involved in the coordinated regulation of the cycling expression of the key CAM gene, *PPCK*. Furthermore, the PIF1/PIF3 were also important TFs involved in photoperiod process (Leivar and Monte, 2014; Zhang et al., 2017), and *HSFA6B*/*HSFA2B* were two cycling heat shock factors in pineapple leaves (Sharma et al., 2017). Interestingly, STOP1, a factor responses to acidic pH and activates a malate efflux transporter in *Arabidopsis* (Iuchi et al., 2007), showed a diurnal expression pattern coincided with the day/night oscillation of malate concentration in pineapple leaf (Sharma et al., 2017). The co-occurrence of the motifs of these TFs involved in differential pathways indicated that the *PPCK* gene might be co-regulated by multiple processes through mediating the cross-talk between the circadian, photoperiod, temperature stress and malate metabolism signaling pathways.

In addition, another two genes, *Aco004996* (*MDH*) and *Aco024818* (*PPDK*), which showed diurnal expression pattern in pineapple green leaf tissues, were also found one DHS in each of the gene’s promoter region exhibiting more accessible at 10 a.m. than 2 a.m. (Figures 6E, F). Furthermore, the motifs of *CDF3* and *CHE* were identified in the DHS related to *Aco004996*, meanwhile motifs of *CHE* and *PIF1*/*PIF3* were found in DHS related to *Aco024818*.

## Discussion

### Genome-wide DHSs features in pineapple leaf tissues

CAM photosynthesis is thought evolved from C3 pathway by reconfiguring gene expression coordinated with circadian (Ming et al., 2015). Therefore, deciphering the regulatory network between the circadian and CAM pathway is important to understand the CAM pathway evolution. By analyzing genome-wide DHSs in pineapple leaves at two timepoints during day and night, we obtained the genome-wide DHSs features in pineapple leaf tissues. The DHSs distribution across the chromosome is consistent with the gene density trends and the majority (70%–80%) of them located in the promoter and intergenic regions (Figures 2B, C). These results are consistent with previous reports in other plant species (Zhang et al., 2012a; Zhang et al., 2012b; Qiu et al., 2016; Lu et al., 2019). However, we found that the promoter DHSs were mostly located within 1 kb upstream of TSS (Figure 2C). In addition, most of the pineapple genes possess less than two DHSs and with length less than 1 kb (Figures 2D, E). These features might be related to the compact gene structure of pineapple genome, 45% of which were intergenic regions (Supplementary Figure S4) and the distance between genes was relatively small, only 9546 bp on average. Furthermore, by comparing the DHS features related to TFs/TCs and those associated with other functional genes, we found that the TFs/TCs tend to have even more and longer DHSs than other functional genes (Figures 3A, B). Importantly, the DHSs associated with TF/TC genes were more preferentially located in the intergenic regions (Figure 3C), indicating a complex regulatory context around TFs/TCs. Previous studies reported that the intergenic DHSs might be functionally related with enhancers or lncRNAs (Qiu et al., 2016; Ricci et al., 2019). Thus, we infer that the TF/TCs regulatory mode is more diverse and complex than other functional genes. Nevertheless, this speculation still needed further confirmation, since this is the first report about DHSs features around TF/TC genes.

### Preferentially accessible DHSs in photosynthetic tissues and the regulatory networks induced by circadian TFs

The circadian rhythm is maintained by a complex regulation of interlocked transcription-translation feedback loops (Zhang and Kay, 2010; Hsu et al., 2013). The clock-coordinated CAM pathway is observed in pineapple. By quantitative comparisons of DHSs between photosynthetic and non-photosynthetic leaf tissues, we identified the preferentially accessible DHSs in photosynthetic leaf tissues and the coordinately changed genes (CCGs) with differential gene expression (Figures 4A, C). GO function of these CCGs were enriched in photosynthesis related pathways (Supplementary Figure S6). These results indicated that the gene expression and chromatin accessibility of photosynthesis pathways related genes were coordinately changed in pineapple leaves. Interestingly, we found circadian clock related TFs were also coordinately regulated in pineapple photosynthetic leaf tissues (Supplementary Datasets S2). Thus, we conducted three regulatory networks between the circadian TFs and the photosynthesis related genes by searching the TF motif occurrences in the DHSs which were specific in AcG2 or AcG10, and those were common accessible both in AcG2 and AcG10 (Figure 5). We found that so many genes were potentially the regulatory targets of circadian TFs in pineapple leaves, including 59 TF genes with cycling expression patterns in pineapple green leaf tissues and many photosynthesis related genes (Supplementary Datasets S3). This result is consistent with the complexity of the circadian oscillator network in *Arabidopsis*, in which one-third of genes were regulated by circadian rhythm through integrating environmental timing cues with the central oscillator and regulating diverse processes by output pathways (Harmer and Kay, 2005), such as growth, development (Blasing et al., 2005; Covington et al., 2008), and response to abiotic and biotic stresses (Goodspeed et al., 2012). Taken together, our results offer valuable information about the gene regulatory landscape in pineapple leaves under light/dark cycles, which may promote further analysis of circadian regulation of CAM photosynthesis.

### The complex and dynamic diel regulation of CAM genes in pineapple leaves

In this study, we surprisingly found that at least one of the 12 clock regulatory motifs in the DHSs nearest to 11 of the CAM genes (Supplementary Datasets S4), indicating a diel and complex regulation of CAM genes. Most strikingly, two key CAM enzyme encoding genes were dynamically regulated by circadian related TFs through co-occurrence of several clock TFs (Figures 6C, D). Circadian regulation of CO_2_ fixation is the key metabolic character that distinguishes CAM from the ancestral C_3_ pathway (Yang et al., 2015; Hartwell et al., 2016). The nocturnal fixation of atmospheric CO_2_ in CAM plants is catalyzed by PEPC (Nimmo, 2003a). Previous studies usually observed no gene expression cycling of the PEPC gene in CAM plants during day/night cycling (Nimmo, 2003b; Ping et al., 2018). The *in vivo* activity of PEPC is determined by its phosphorylation status, which is catalyzed by the kinase PPCK in CAM plants (Hartwell et al., 1999; Taybi et al., 2000; Boxall et al., 2017). However, we identified a PEPC encoding gene (*Aco010025*) which not only shows a dynamic expression pattern in pineapple leaves, but also exhibited a dynamic DHS in the promoter region containing *cis*-regulatory elements can be identified by clock related TFs, such as *CCA1*, *CHE*, and *CDF2/CDF3* (Figures 6B, C). This might be a distinct feature of the CAM pathway in pineapple genome, deserving further studies. PPCK (*Aco013938*) is another key enzyme in CAM pathway. The circadian rhythm of PPCK transcripts and activity under constant conditions reveled that its activity is regulated by a circadian clock (Hartwell et al., 1999; Nimmo, 2000). It is argued that the identification of key regulators of *PPCK* will be critical for further understanding the circadian regulation of CAM photosynthesis (Hartwell, 2005). By comparing the DHSs at the light and dark timepoints, we identified two adjacent DHSs in the promoter region and gene body, respectively. The one located in the promoter region showed more accessible at 10 a.m. than 2 a.m., containing *cis*-regulatory sequences identified by *PIF1*, *PIF3*, *CHE* and *CDF2*, and the other one was more accessible at 2 a.m. than 10 a.m., containing sequences can be identified by *CDF3*, *STOP1* and *HSFA6B*/*HSFA2B* (Figure 6D). The motifs occurred in these DHSs nearby CAM genes could be recognized by TFs involved in multiplex pathways, including PIF1/PIF3 which was in photoperiod process (Leivar and Monte, 2014; Zhang et al., 2017), *HSFA6B*/*HSFA2B* which was response to heat shock (Sharma et al., 2017), and STOP1 which can respond to acidic pH and activate a malate efflux transporter (Iuchi et al., 2007). Therefore, the co-occurrence of multiple motifs in the DHSs of CAM genes indicated a complex co-regulation of CAM pathway in pineapple through cross-talk between the circadian, photoperiod, temperature stress and malate metabolism signaling pathways. In short, our results indicate that the DHS-based approach is practical for mapping the *cis*-regulatory landscape of diel control of CAM photosynthesis genes which could provide essential information for achieving a more detailed understanding of the evolutionary pathway from C3 to CAM.

## Conclusion

Open chromatin regions also called DNase I hypersensitive sites (DHSs) are usually associated with *cis*-regulatory elements (CREs). Here we developed a simplified DNase-seq method to mapping the chromatin regulatory landscape in pineapple leaves during day and night. Our result suggested that several cycling TFs binding motifs were identified in DHSs nearby several key CAM genes, strongly confirming the circadian regulation of CAM pathway in pineapple. This result will help to deciphering the molecular mechanism of diel regulation of CAM.

## Data Availability

The pineapple DNase-seq data sets have been deposited to the Sequencing Read Archive (SRA) at the National Center for Biotechnology Information (NCBI), under the BioProject accession number of PRJNA756386 [https://www.ncbi.nlm.nih.gov/bioproject/PRJNA756386/]. The remaining datasets presented in this study can be found in Supplementary Material.

## Supplementary material

SUPPLEMENTARY FIGURE S1 DNase-seq data correlation among three biological replicates of *Arabidopsis* samples.

SUPPLEMENTARY FIGURE S2 DNase-seq data correlation among three biological replicates of four pineapple leaf samples.

SUPPLEMENTARY FIGURE S3 GO enrichment analysis of preferentially expressed genes in AcG2, AcW2, AcG10 and AcW10.

SUPPLEMENTARY FIGURE S4 GO enrichment analysis of the coordinately changed genes in AcG2, AcW2, AcG10 and AcW10.

SUPPLEMENTARY FIGURE S5 GO enrichment analysis of genes with more accessible DHSs in AcG2 and AcG10, respectively.

SUPPLEMENTARY FIGURE S6 The regulatory network between the coordinately changed genes.

SUPPLEMENTARY FIGURE S7 The heatmap of CAM involved genes in photosynthetic and non-photosynthetic tissues.

SUPPLEMENTARY TABLE S1 Summary of the DNase-seq data of the four pineapple samples.

SUPPLEMENTARY TABLE S2 The percentages of intergenic DHSs from top 10% TFs/TCs with most number or length of DHSs.

SUPPLEMENTARY DATASETS S1 The final peaks identified in each of the pineapple leaf tissues.

SUPPLEMENTARY DATASETS S2 The list of DDHSs, DEGs and coordinately changed genes.

SUPPLEMENTARY DATASETS S3 The potential regulatory relationship between the coordinately changed genes.

SUPPLEMENTARY DATASETS S4 The clock related motif occurrences in DHSs around 11 diurnally expressed CAM genes.

## Abbreviations

CAM, Crassulacean acid metabolism; DHSs, DNase I hypersensitive sites; CREs, cis-regulatory elements; TSS, transcription start site; TES, transcription end site; AcG2, green-tip tissue at 2 a.m.; AcG10, green-tip tissue at 10 a.m.; AcW2, white-base tissue at 2 a.m.; AcW, 10white-base tissue at 10 a.m.; DDHSs, different DHSs; DEGs, differentially expressed genes; CCGs, coordinately changed genes.
