# Supplementary material for: Genome-wide mapping of DNase I hypersensitive sites in pineapple leaves
Source: Front Genet. 2023 Jul 4;14:1086554. doi: 10.3389/fgene.2023.1086554 (PMC10352800; doi:10.3389/fgene.2023.1086554)
Supplement: Supplementary file 2 [file Table1.docx]

**Supplemental Table S1 Summary of the DNase-seq data of the four pineapple samples.**

| Samples | Clean reads | Uniqe reads | rm_organelle | <125bp | DHSs (IDR) |
| --- | --- | --- | --- | --- | --- |
| AcG2-1 | 102400075 | 38829584 | 35950111 | 26021285 | 33340 |
| AcG2-2 | 169325603 | 62012113 | 57464483 | 38854339 |  |
| AcG2-3 | 141280257 | 54927844 | 51093600 | 34012240 |  |
|  |  |  |  |  |  |
| AcW2-1 | 150645289 | 58132005 | 44348505 | 21041404 | 29597 |
| AcW2-2 | 129417720 | 50829041 | 39862577 | 21088323 |  |
| AcW2-3 | 126327086 | 44175845 | 35062086 | 17245006 |  |
|  |  |  |  |  |  |
| AcG10-1 | 152072367 | 52875856 | 46579575 | 21240240 | 28753 |
| AcG10-2 | 162913245 | 59076528 | 52389639 | 24686675 |  |
| AcG10-3 | 139255234 | 49628780 | 44164400 | 24902266 |  |
|  |  |  |  |  |  |
| AcW10-1 | 176576974 | 85042014 | 70696985 | 31801180 | 40068 |
| AcW10-2 | 202610462 | 101427697 | 85049579 | 39469958 |  |
| AcW10-3 | 172048790 | 85851736 | 73799664 | 38413602 |  |
