# Supplementary material for: Genome-wide mapping of DNase I hypersensitive sites in pineapple leaves
Source: Front Genet. 2023 Jul 4;14:1086554. doi: 10.3389/fgene.2023.1086554 (PMC10352800; doi:10.3389/fgene.2023.1086554)
Supplement: Supplementary file 4 [file Table2.docx]

**Supplemental Table S2 The percentages of intergenic DHSs from top 50% TFs/TCs or non-TF/TC genes with most number or length of DHSs.**

| Samples | top 50% TFs/TCs with most number of DHSs | top 50% TFs/TCs with most length of DHSs | top 50% non-TF/TC genes with most number of DHSs | top 50% non-TF/TC genes with most length of DHSs |
| --- | --- | --- | --- | --- |
| AcG2 | 47.63 | 47.58 | 39.34 | 36.89 |
| AcG10 | 37.84 | 37.81 | 34.60 | 32.46 |
| AcW2 | 56.32 | 55.94 | 45.18 | 46.13 |
| AcW10 | 52.40 | 53.23 | 45.89 | 45.10 |
