# Supplementary material for: Genome-wide mapping of DNase I hypersensitive sites in pineapple leaves
Source: Front Genet. 2023 Jul 4;14:1086554. doi: 10.3389/fgene.2023.1086554 (PMC10352800; doi:10.3389/fgene.2023.1086554)
Supplement: Supplementary file 7 [file DataSheet1.docx]

Supplemental Figures


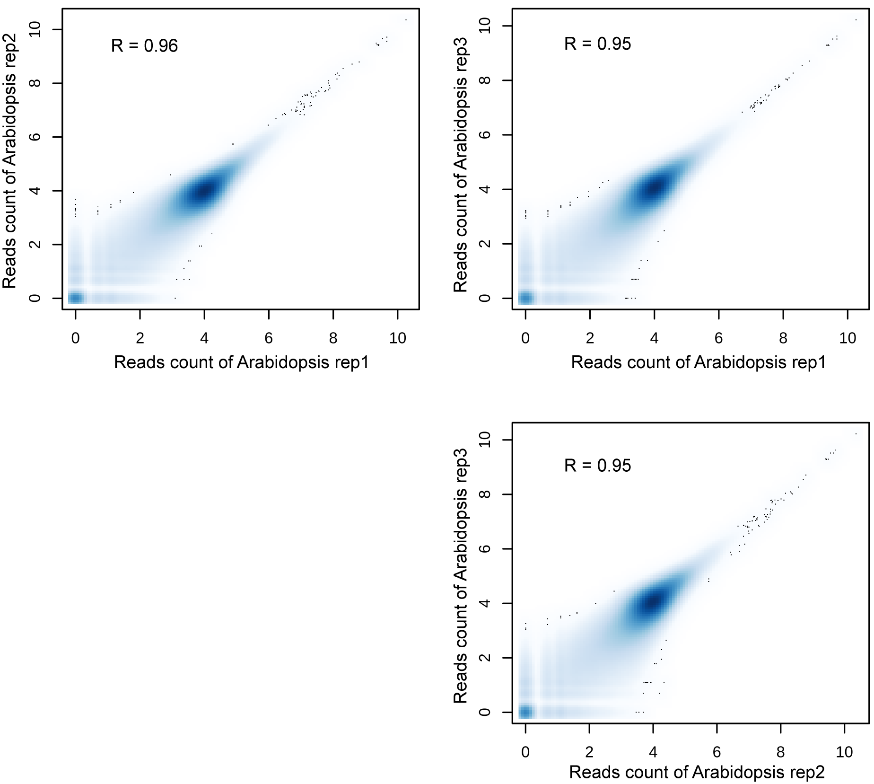


**Supplemental Figure S1. DNase-seq data correlation among three biological replicates of *Arabidopsis* samples.**

The entire *Arabidopsis* genome was divided into 100 bp non-overlapping windows. The number of DNase-seq reads in the windows were used to calculate the Pearson Correlation Coefficient between replicates.


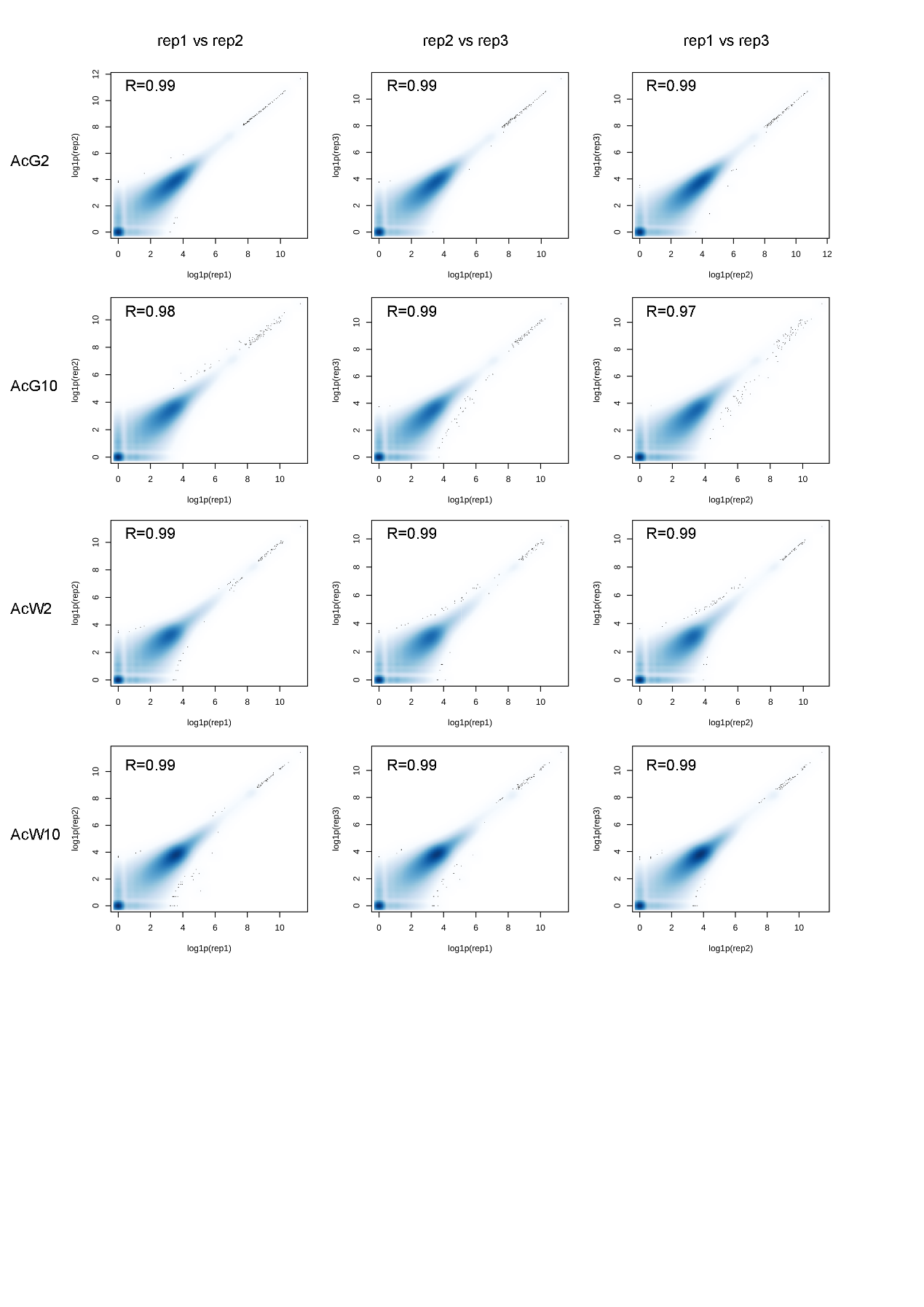


**Supplemental Figure S2. DNase-seq data correlation among three biological replicates of four pineapple leaf samples.**

The entire pineapple genome was divided into 100 bp non-overlapping windows. The number of DNase-seq reads in the windows were used to calculate the Pearson Correlation Coefficient between replicates.


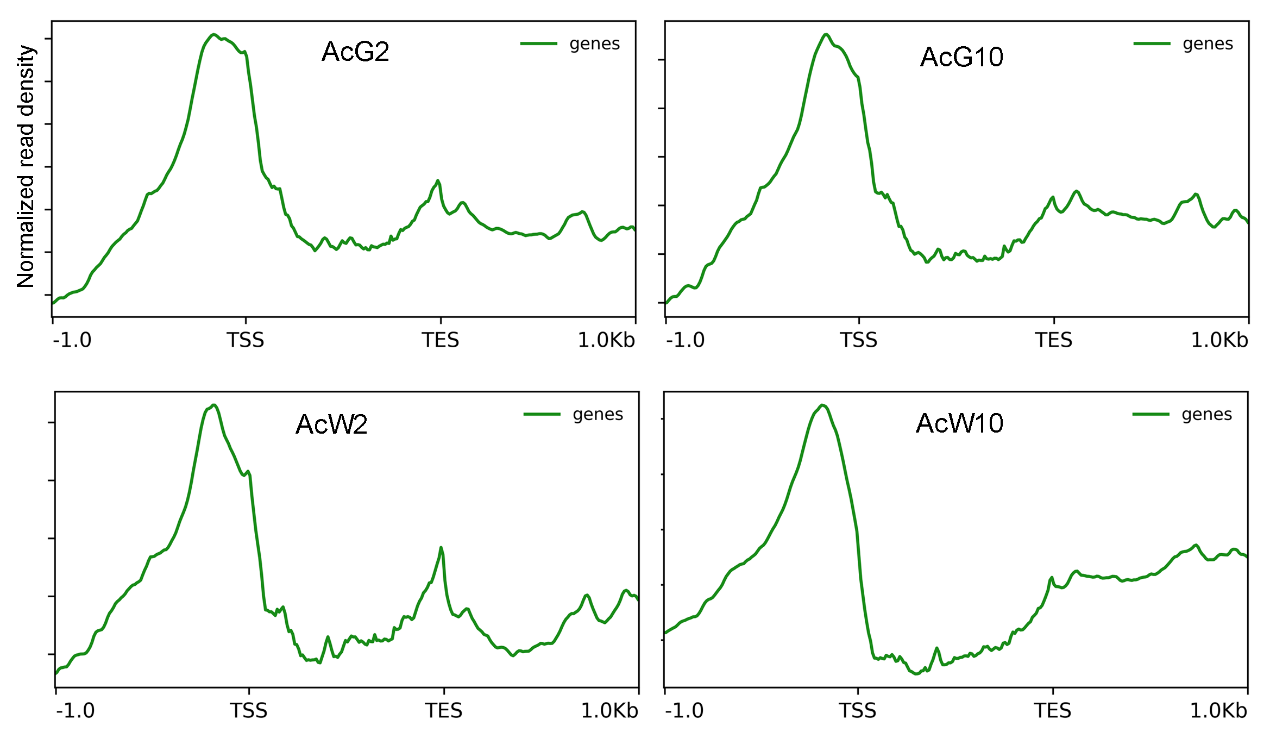


**Supplemental Figure S3** The distribution of DNase-seq reads in pineapple samples.

**Supplemental Figure S4 The distribution of DHSs length relative to different gene regions in each sample.** AcG2, AcG10, AcW2 and AcW10 were pineapple samples indicating the percentage of total DHS length assigned to different locations; while *Genome* indicated the ratio of seven sequence classes in pineapple genome.


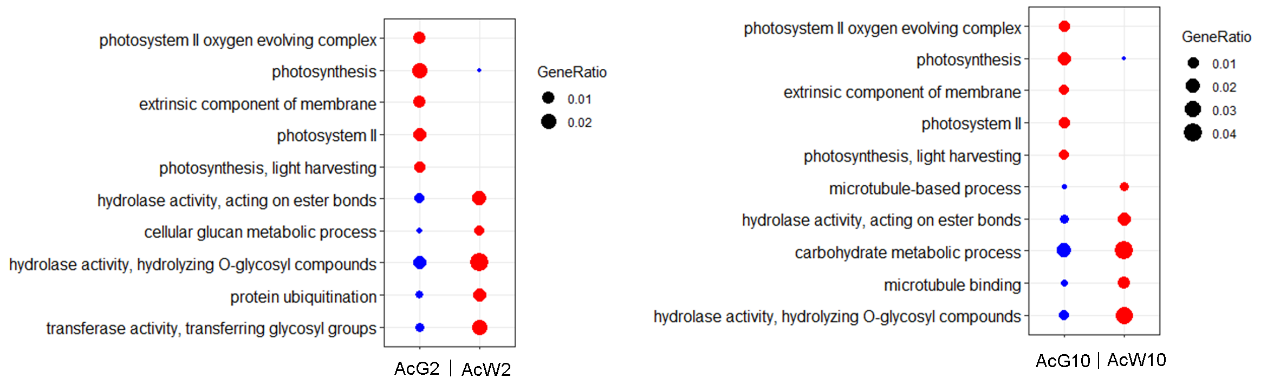


**Supplemental Figure S5 GO enrichment analysis of preferentially expressed genes in AcG2, AcW2, AcG10 and AcW10.**


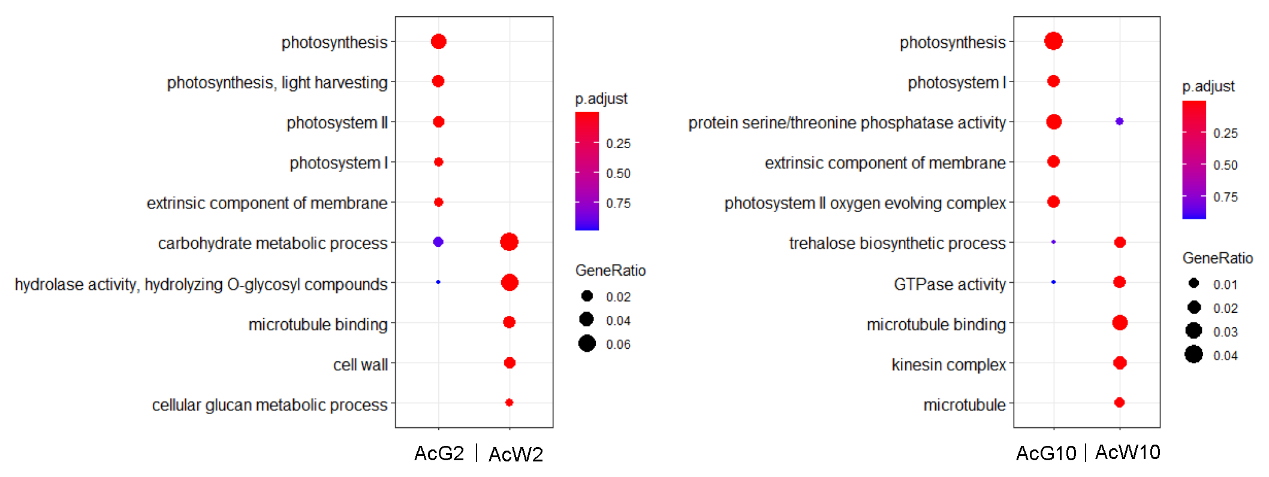


**Supplemental Figure S6. GO enrichment analysis of the coordinately changed genes in AcG2, AcW2, AcG10 and AcW10.**


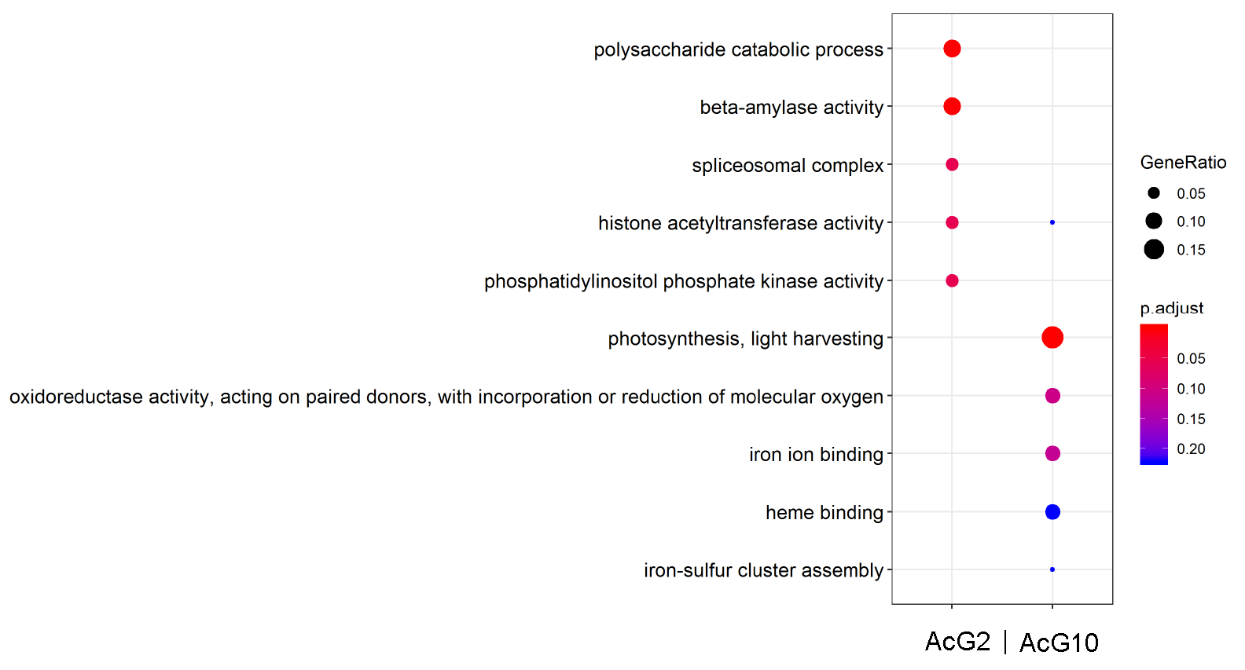


**Supplemental Figure S7. GO enrichment analysis of genes with more accessible DHSs in AcG2 and AcG10, respectively.**


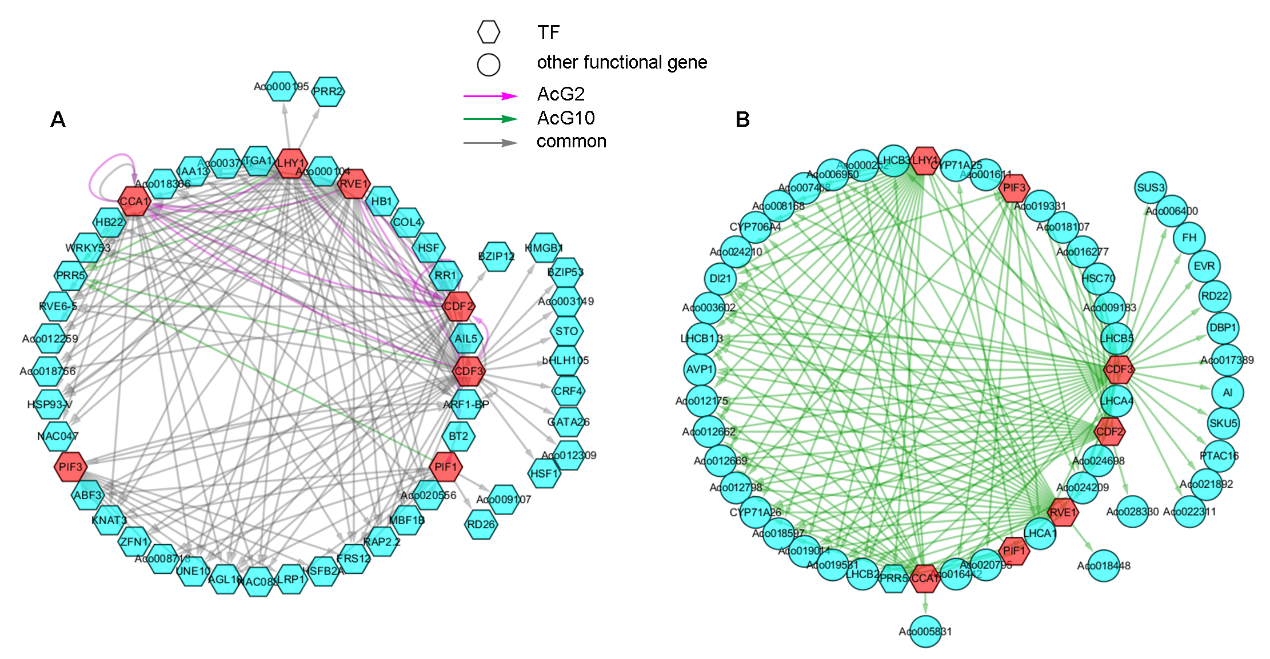


**Supplemental Figure S8. The regulatory network between the coordinately changed genes.**

**A.** The regulatory network between genes with only AcG10-sepcific DHSs. **B.** The regulatory network between TF genes with common accessible DHSs in AcG2 and AcG10. The clock related TFs were colored in red and the potential targets in blue. TFs were showed as hexagons and other functional genes as circles.


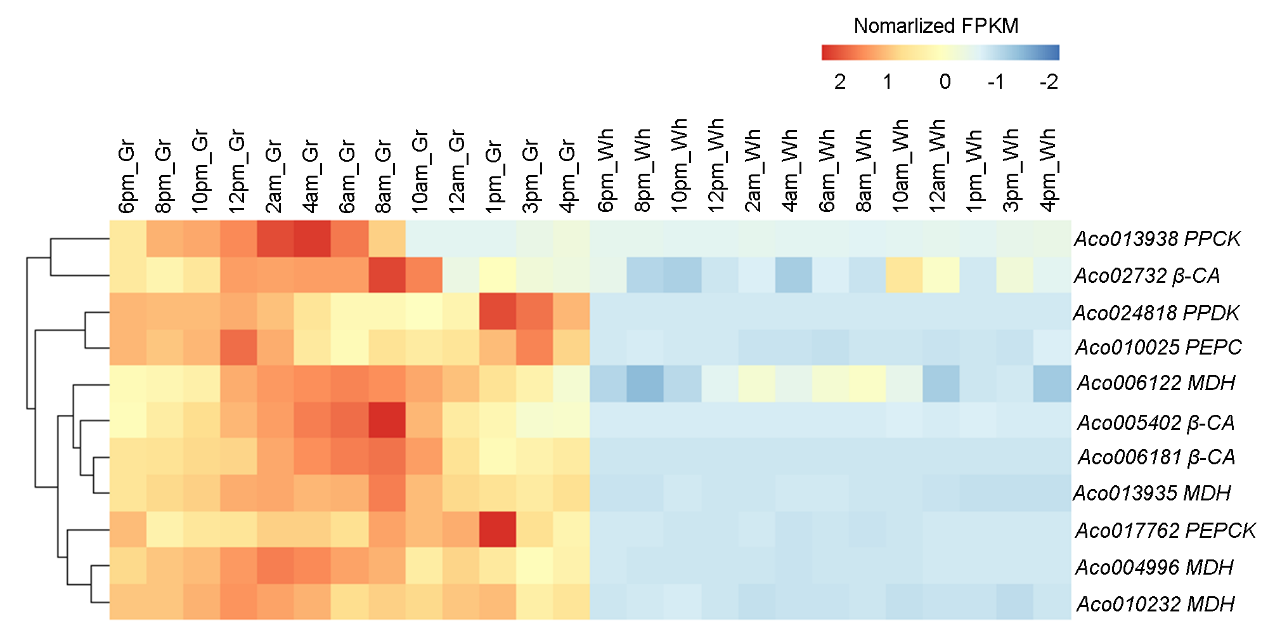


**Supplemental Figure S9. The heatmap of CAM involved genes in photosynthetic and non-photosynthetic tissues.**

These genes exhibited high expression level in photosynthetic (green tip, Gr) but low or no expression in non-photosynthetic (white base, Wh) leaf tissues. The genes were clustered based on gene expression level, red indicated high expression level and blue indicated low expression level.
